# The dose–response relationship between smoking and the risk factor for invasive pulmonary aspergillosis in patients with severe fever with thrombocytopenia syndrome

**DOI:** 10.3389/fmicb.2023.1209705

**Published:** 2023-06-30

**Authors:** Yan Dai, Qinqin Pu, Nannan Hu, Jin Zhu, Yaping Han, Ping Shi, Jun Li, Ke Jin

**Affiliations:** ^1^Department of Infectious Disease, The First Affiliated Hospital of Nanjing Medical University, Nanjing, China; ^2^Epidemiological Department, Huadong Medical Institute of Biotechniques, Nanjing, China

**Keywords:** severe fever with thrombocytopenia syndrome, invasive pulmonary aspergillosis, smoking, risk factors, infection

## Abstract

**Objectives:**

Invasive pulmonary aspergillosis (IPA) is common in immuno-compromised people, and a high incidence of IPA has been found in patients with severe fever with thrombocytopenia syndrome (SFTS). Our study aimed to determine the independent risk factors for IPA and the relationship between smoking status and the risk of IPA in SFTS patients.

**Methods:**

A retrospective analysis of SFTS patients in the First Affiliated Hospital of Nanjing Medical University from May 2011 to December 2021 was reviewed. The patients were divided into two groups: IPA and non-IPA groups. We compared demographic characteristics, clinical manifestation, laboratory parameters, treatment, and prognosis, and explored the risk factors of IPA using logistic regression and ROC curve. The dose-dependent effect of smoking on the risk of IPA was further estimated, including the age of smoking initiation, daily smoking amount, smoking duration, and pack-years of smoking.

**Results:**

In total, 189 individuals were included. Compared with the non-IPA group, the IPA group had higher levels of smoking, drinking, cough, dyspnea, aCCI scores, Dabie bandavirus (DBV) RNA load, ferritin, PCT, IL-6, APTT, LDH, BUN, creatinine, and lower levels of FT4 and TSH. The incidences of MODS, admission to ICU, ventilation, and broad-spectrum antibiotic treatment were significantly higher in the IPA group than in the non-IPA group. Multivariable logistic analysis showed that smoking history, cough, creatinine, admission to ICU, broad-spectrum, and corticosteroid therapies were the independent risk factors for IPA in SFTS patients. We further confirmed that the age of smoking initiation <30 years, smoking at least one pack per day, smoking for at least 40 years, and having at least 40 pack-years of smoking exposure were the independent risk factors for IPA among smokers.

**Conclusion:**

The prognosis of SFTS patients in the IPA group is worse than that of the non-IPA group. Attention should be paid to SFTS patients with a smoking history, cough, creatinine, admission to ICU, and broad-spectrum and corticosteroid therapies. There is a strong dose-dependent association between smoking and IPA development in SFTS patients. Prophylactic antifungal therapy should be considered for SFTS patients with these risk factors, but further studies are necessary to determine if it is beneficial for the prognosis of these patients.

## Introduction

Severe fever with thrombocytopenia syndrome (SFTS) has been known as a severe tick-borne disease that is becoming a global disease and was first identified in 2009 in China (Yu et al., 2011). It is caused by Dabie bandavirus (DBV). The principal signs of this hemorrhagic fever disease are fever, thrombocytopenia, leukopenia, and gastrointestinal tract symptoms, while in severe cases, the syndrome is often complicated by multiple organ dysfunction syndrome (MODS) and shock, which eventually lead to a high case fatality rate (CFR). The CFR of SFTS ranges between 16.2 and 35% when broken down by region (Li et al., 2018; Yun et al., 2020; Chen et al., 2022; Yokomizo et al., 2022). While SFTS has been seen as a threat to public health, there is no effective medicine to treat it beyond symptomatic treatment (Zhang et al., 2021).

A significantly high rate of coinfection with other pathogens in SFTS patients has been reported in several current studies as a result of systemic inflammatory response syndrome (SIRS) and immunologic derangement. Invasive pulmonary aspergillosis (IPA), which is thought to be one of the most frequent infections in SFTS patients, widely develops in immuno-compromised people and has been found to increase the mortality of SFTS according to several studies (Sakaguchi et al., 2019; Iwao et al., 2021; Ge et al., 2022). The incidence of IPA in SFTS is ~20%, while the mortality of these cases is between 20 and over 50% (Bae et al., 2020; Hu et al., 2021; Xu et al., 2021; Zhang et al., 2022). A study from China reported that patients who received early antifungal treatment tended to have improved survival rates, which indicated the necessity of early diagnosis and timely treatment (Hu et al., 2021). However, the risk factors associated with IPA development in SFTS patients were rarely investigated. This study systematically assessed the impact that different potential factors have on the risk for IPA development in SFTS patients.

Based on the present studies, cigarette smoking is a major preventable environmental factor and plays a strong part in the suppression of the immune system, which partly leads to a high frequency of IPA in several diseases (Calderón-Parra et al., 2022; Shi et al., 2022). However, there is no specific evidence to support the relationship between smoking and the development of IPA in SFTS patients thus far, mostly due to limited data. Due to its high mortality, analyses should be performed to investigate whether smoking might have affected the incidence of IPA in SFTS cases. Consequently, we further focused on the association between smoking and IPA in SFTS.

## Methods

### Study design and participants

For this retrospective study, SFTS patients who were admitted to the First Affiliated Hospital of Nanjing Medical University from May 2011 to December 2021 were included. Patients who died on the day of admission or had missing clinical data were excluded. The study protocol was approved by the responsible ethics committee (Protocol No. 2022-SR-366).

### Definitions of SFTS and IPA

The diagnosis of SFTS was based on the criteria published by the Chinese Ministry of Health (Ministry of Health, 2011). The guideline divided SFTS diagnoses into suspected and confirmed diagnoses. This study included only patients with confirmed SFTS diagnosis. The confirmed diagnosis was defined as patients who met the first three criteria and were tested to meet one of the last four criteria: (1) epidemiological risk factors (including a history of bite from a tick and contact with hilly areas in epidemic seasons); (2) acute fever, fatigue, or other clinical manifestations; (3) thrombocytopenia and leukocytopenia; (4) positive for DBV RNA using polymerase chain reaction; (5) seroconversion in IgG/IgM antibodies to DBV; (6) IgG antibodies to DBV were detected more than four times in samples during the convalescent stage compared with the samples during the acute stage; and (7) the isolation of DBV from the samples.

The diagnosis of IPA was based on the criteria published by the European Organization for the Research and Treatment of Cancer/Mycosis Study Group (EORTC/MSG) (Donnelly et al., 2020). The guideline divided IPA diagnoses into proven, probable, and possible diagnoses. Patients with proven and probable diagnoses were included in our study. The proven diagnosis was based on the following criteria: (1) microscopic examination of the samples proved the growth of Aspergillus hyphae, (2) the positive culture of *Aspergillus* spp. from a normally sterile and clinically or radiologically abnormal site, excluding bronchoalveolar lavage (BAL) fluid, and (3) amplifying the Aspergillus DNA and sequencing the PCR-amplified products. The diagnosis was considered probable if the patients have appropriate host factors, clinical manifestations, and mycological evidence.

### Data collection

All the information was collected from the electronic hospital records system. The following data from each patient were reviewed: demographic data, clinical manifestation, laboratory parameters, treatment (the use of broad-spectrum antibiotics and corticosteroids), and prognosis. Comorbidity was categorized by the age-adjusted Charlson comorbidity index (aCCI). All participants were divided into three groups: current smokers, past smokers, and never smokers. We defined current smokers as patients who had smoked more than 100 cigarettes and had smoked in the month before IPA development. Past smokers were defined as patients who had smoked more than 100 cigarettes but had quit smoking in the month before IPA development. Never smokers were defined as patients who had smoked <100 cigarettes or had never smoked. The first two classifications were collected and referred to as ever smokers. We also gathered detailed information about smoking, including the age of smoking initiation, daily smoking amount, smoking duration, and pack-years of smoking (the number of packs of cigarettes per day × the years of smoking).

### Statistical analysis

All statistical analyses were performed using IBM SPSS Statistics version 26.0 (IBM Corp., Armonk, NY, United States) or GraphPad Prism 9.0 (GraphPad Software, San Diego, United States). Continuous variables were expressed as mean ± standard deviation (SD) or median with interquartile range (IQR), and categorical variables were expressed as frequency and percentage. The *t*-test or Mann–Whitney *U*-test was used to compare the continuous variables if appropriate, while the chi-square test was used to compare the categorical variables. We used univariate and multivariate logistic regression analyses to calculate the odds ratios (ORs) and 95% confidence intervals (CIs) for the risk factors associated with the development of IPA. We further used logistic regression models to analyze the adjusted risk of IPA development based on the age of smoking initiation, daily smoking amount, smoking duration, and pack-years of smoking, and the results were also described as ORs and 95% CIs. Survival analysis comparisons between the IPA and non-IPA groups were analyzed by the log-rank (Mantel–Cox) test and Kaplan–Meier curves with the 30-day follow-up period. Receiver operating characteristic (ROC) curves were plotted to assess the predictive performance of several indexes with the calculation of the area under the curve (AUC). All tests were two-tailed, and a *P-*value of <0.05 was considered statistically significant.

## Results

### Population characteristics and baseline clinical information

A total of 189 individuals were analyzed in this study with a mean age of 63.16 ± 11.38 years. Among them, 39 (20.6%) were included in the IPA group and 150 (79.4%) were included in the non-IPA group. Comparisons of demographics, clinical manifestation, laboratory parameters, and other clinical information are presented in Table 1. IPA patients had a mean age of 65.59 ± 7.78 years, and 66.7% were men, while the non-IPA group had a mean age of 62.53 ± 12.09 years, and 49.3% were men. The IPA group had significantly higher aCCI scores than the non-IPA group (*P* < 0.05). Among the 39 IPA patients, 18 (46.2%) were current smokers, 2 (5.1%) were past smokers, 19 (48.7%) were never smokers, and 16 (41.0%) were drinkers, and the proportions of them were significantly higher in the IPA group than in the non-IPA group (*P* < 0.05).

**Table 1:** Characteristics between IPA and non-IPA patients with severe fever with thrombocytopenia syndrome.

**Factors**	**Total (*n* = 189)**	**Non-IPA (*n* = 150)**	**IPA (*n* = 39)**	***P*-value**
**Demographics**				
Age, years	63.16 ± 11.38	62.53 ± 12.09	65.59 ± 7.78	0.057
Male, *n* (%)	100 (52.9)	74 (49.3)	26 (66.7)	0.053
Dead, *n* (%)	57 (30.2)	36 (24.0)	21 (53.8)	0.000
aCCI	3.0 (2.0–4.0)	3.0 (2.0–4.0)	4.0 (3.0–4.0)	0.032
**Smoking history**, ***n*** **(%)**				
Current smoking	40 (21.2)	22 (14.7)	18 (46.2)	0.000
Past smoking	6 (3.2)	4 (2.7)	2 (5.1)	
Never smoking	143 (75.7)	124 (82.7)	19 (48.7)	
Alcohol consumption, *n* (%)	38 (20.1)	22 (14.7)	16 (41.0)	0.000
**Clinical manifestation**				
Cough, *n* (%)	72 (38.1)	49 (32.7)	23 (59.0)	0.003
Dyspnea, *n* (%)	44 (23.3)	29 (19.3)	15 (38.5)	0.012
**Laboratory parameters**				
RNA load (lg copies/mL)	6.32 (5.96–6.61)	6.32 (5.95–6.38)	6.48 (6.07–7.16)	0.019
WBC count (× 10^9^/L)	3.14 (1.58–6.02)	2.94 (1.59–5.74)	4.31 (1.55–7.16)	0.217
Neutrophil count (× 10^9^/L)	2.01 (0.96–4.31)	1.86 (0.94–4.23)	2.80 (1.11–4.97)	0.286
Lymphocyte count (× 10^9^/L)	0.66 (0.42–1.24)	0.65 (0.41–1.19)	0.8 (0.45–1.40)	0.386
Monocyte count (× 10^9^/L)	0.14 (0.08–0.35)	0.15 (0.08–0.34)	0.12 (0.07–0.42)	0.894
Hemoglobin (g/L)	131.82 ± 22.22	130.35 ± 22.14	137.46 ± 21.90	0.077
Platelet count (× 10^9^/L)	39.00 (28.00–55.00)	39.00 (27.00–55.25)	39.00 (29.00–53.00)	0.589
CD3^+^CD4^+^ cells (/μL)	191.50 (123.00–312.50)	198.00 (121.50–343.75)	183.50 (122.25–232.00)	0.285
CD3^+^CD8^+^ cells (/μL)	206.00 (116.25–447.25)	213.00 (112.75–519.00)	179.50 (111.00–342.50)	0.539
CD3^−^CD19^+^ (/μL)	155.50 (61.00–279.50)	148.50 (58.00–292.00)	175.00 (119.25–269.00)	0.601
CD3^−^CD56^+^ (/μL)	124.50 (75.25–206.00)	124.50 (79.50–206.00)	115.00 (69.25–268.00)	0.844
CD3^+^CD56^+^ (/μL)	13.95 (12.14–33.21)	13.95 (13.19–26.47)	17.08 (7.63–49.26)	0.873
CD3^+^CD69^+^ (/μL)	45.83 (28.51–80.02)	41.15 (25.12–145.75)	49.56 (29.56–62.00)	0.690
CD3^+^CD25^+^ (/μL)	58.37 (27.40–106.64)	60.64 (27.51–131.08)	50.30 (26.68–74.69)	0.328
CD3^+^HLADR^+^ (/μL)	158.28 (86.38–299.52)	156.58 (84.69–313.78)	159.97 (88.22–294.77)	0.847
FERR (ng/mL)	8,577.15 (1,536.93–15,000.00)	6,962.55 (1,500.00–15,000.00)	15,000.00 (6,665.37–18,187.50)	0.001
PCT (ng/mL)	0.27 (0.13–0.55)	0.23 (0.12–0.54)	0.35 (0.21–0.76)	0.022
IL-6 (ng/mL)	0.05 (0.02–0.12)	0.02 (0.01–0.09)	0.10 (0.05–0.18)	0.001
PT (s)	12.20 (11.50–13.00)	12.20 (11.50–13.00)	12.10 (11.60–12.90)	0.996
APTT (s)	45.30 (38.90–56.33)	44.20 (38.35–53.55)	49.00 (42.40–63.30)	0.026
D-Dimer (mg/L)	2.59 (1.31–7.79)	2.54 (1.24–6.58)	2.70 (1.65–9.39)	0.593
ALT (U/L)	111.05 (60.70–168.78)	102.90 (61.25–162.10)	129.90 (60.60–172.20)	0.469
AST (U/L)	318.60 (165.75–544.68)	298.20 (152.45–506.15)	365.10 (232.00–650.70)	0.059
ALP (U/L)	74.00 (55.85–114.98)	71.00 (54.00–112.00)	87.00 (60.50–132.95)	0.159
GGT (U/L)	68.30 (28.50–144.30)	65.85 (27.35–135.00)	81.00 (42.85–225.10)	0.100
LDH (U/L)	1,107.00 (675.50–1,749.00)	989.00 (599.00–1,544.00)	1,489.00 (1,040.00–2,643.50)	0.001
ADA (U/L)	41.40 (27.78–63.53)	39.80 (27.83–62.98)	47.55 (27.30–69.58)	0.307
Albumin (g/L)	30.35 ± 4.81	30.49 ± 4.88	29.83 ± 4.58	0.446
BUN (mmol/L)	5.91 (4.33–8.15)	5.42 (4.05–7.62)	7.70 (5.48–11.69)	0.000
Creatinine (μmol/L)	68.00 (56.83–89.88)	65.90 (53.90–87.40)	88.10 (66.60–125.10)	0.000
Pro-BNP (pg/mL)	973.10 (386.60–4,024.00)	924.70 (300.90–4,091.50)	1,121.00 (703.45–4,221.65)	0.090
FT3 (pmol/L)	2.22 ± 0.86	2.28 ± 0.91	1.90 ± 0.46	0.064
FT4 (pmol/L)	12.34 (10.89–15.53)	12.85 (11.03–15.95)	10.81 (8.85–13.64)	0.043
TSH (mIU/L)	0.72 (0.29–2.21)	1.10 (0.38–2.38)	0.35 (0.11–0.68)	0.019
**Additional factors**				
MODS, *n* (%)	62 (32.8)	35 (23.3)	27 (69.2)	0.000
ICU admission, *n* (%)	58 (30.7)	29 (19.3)	29 (74.4)	0.000
Machined ventilation, *n* (%)	30 (15.9)	15 (10.0)	15 (38.5)	0.000
**Treatment**				
Broad-spectrum antibiotic therapy, *n* (%)	50 (26.5)	24 (16.0)	26 (66.7)	0.000
Corticosteroid use, *n* (%)	98 (51.9)	68 (45.3)	30 (76.9)	0.000
< 1 mg/kg	67 (35.4)	54 (36.0)	13 (33.3)	0.000
≥1 mg/kg	31 (16.4)	14 (9.3)	17 (43.6)	
≤ 5 d	59 (31.2)	42 (28.0)	17 (43.6)	0.002
>5 d	39 (20.6)	26 (17.3)	13 (33.3)	
Never	91 (48.1)	82 (54.7)	9 (23.1)	

In terms of clinical manifestations, 38.1% of the patients presented with cough, and 23.3% of them presented with dyspnea. The proportion of SFTS cases with cough or dyspnea in the IPA group was significantly higher than in the non-IPA group (*P* < 0.05).

As shown in Table 1, the median level of DBV RNA load was significantly higher in the IPA group (*P* < 0.05). The median levels of white blood cell (WBC) count and platelet count were reduced, respectively, and the median levels of neutrophils, lymphocytes, and monocytes were maintained within the normal range. The median levels of inflammatory markers including serum ferritin, procalcitonin (PCT), interleukin-6 (IL-6), and lactate dehydrogenase (LDH) were 8,577.15 ng/ml (interquartile range, 1,536.93–15,000.00), 0.27 ng/ml (interquartile range, 0.13–0.55), 0.05 ng/ml (interquartile range, 0.02–0.12), and 1,107.00 U/L (interquartile range, 675.50–1,749.00), respectively, which were elevated in all patients. The levels of ferritin, PCT, IL-6, and LDH in the IPA group were significantly higher than those in the non-IPA group (*P* < 0.05). With respect to coagulative indicators, the level of activated partial thromboplastin time (APTT) increased in all patients and was higher in the IPA group (*P* < 0.05). There were significant differences in blood urea nitrogen (BUN) and creatinine between the two groups, while other biochemical indexes were not significantly different. In addition, patients who developed IPA had significantly lower free triiodothyronine (FT4) and thyroid-stimulating hormone (TSH) levels than those who did not develop IPA (*P* < 0.05).

The incidences of MODS were 69.2% in the IPA group and 23.3% in the non-IPA group with significant differences (*P* < 0.05). The proportion of admission to the intensive care unit (ICU) was significantly higher in the IPA group (*P* < 0.05). Furthermore, it was shown that the proportions of IPA patients who were treated with machined ventilation and broad-spectrum antibiotic were significantly higher than non-IPA patients. There was also a significant difference in the dose of corticosteroid administered and the length of time it was taken between the two groups. Compared with non-IPA cases, systemic corticosteroid use was more common in IPA cases, and the rates of IPA cases who were treated with a large dose (≥1 mg/kg) and long duration (>5 d) were statistically higher in the IPA group than in the non-IPA group (*P* < 0.05).

### Risk factors for the development of IPA in SFTS patients

A univariate analysis was adopted to test if the significant indexes in Table 1 correlated with the development of IPA. The results of the potential risk factors for IPA development are presented in Table 2. Multivariate logistic regression analysis showed that the following several significant risk factors remained: current smoking (OR 3.764, 95% CI 1.271–11.147, *P* = 0.017), cough (OR 3.501, 95% CI 1.258–9.739, *P* = 0.016), creatinine (OR 1.012, 95% CI 1.003–1.022, *P* = 0.010), ICU admission (OR 4.100, 95% CI 1.439–11.682, *P* = 0.008), a combination of several broad-spectrum antibiotic therapies (≥2 drugs) (OR 5.888, 95% CI 2.095–16.543, *P* = 0.001), use of corticosteroid (OR 4.631, 95% CI 1.499–14.312, *P* = 0.008), small dose use of corticosteroid (< 1 mg/kg) (OR 4.455, 95% CI 1.207–16.451, *P* = 0.025), large dose use of corticosteroid (≥1 mg/kg) (OR 4.819, 95% CI 1.290–18.010, *P* = 0.019), short-term use of corticosteroid (≤ 5 d) (OR 3.833, 95% CI 1.134–12.956, *P* = 0.031), and long-term use of corticosteroid (>5 d) (OR 6.470, 95% CI 1.638–25.556, *P* = 0.008).

**Table 2:** Univariate and multivariate logistic analyses.

	**Univariate analysis**		**Multivariate analysis**	
	**OR (95% CI)**	***P*** **-value**	**OR (95% CI)**	***P*** **-value**
aCCI	1.313 (1.009–1.709)	0.043		
Smoking history		0.000		0.042
Current smoking	5.340 (2.428–11.744)	0.000	3.764 (1.271–11.147)	0.017
Past smoking	3.263 (0.559–19.057)	0.189	3.796 (0.517–27.876)	0.190
Alcohol consumption	4.047 (1.851–8.848)	0.000		
Cough	2.963 (1.437–6.109)	0.003	3.501 (1.258–9.739)	0.016
Dyspnea	2.608 (1.217–5.586)	0.014		
RNA load	1.602 (1.136–2.258)	0.007		
FERR	1.000 (1.000–1.000)	0.098		
PCT	1.033 (0.933–1.144)	0.526		
IL-6	2.363 (0.573–9.749)	0.234		
APTT	1.021 (0.999–1.042)	0.057		
LDH	1.000 (1.000–1.000)	0.866		
BUN	1.139 (1.059–1.226)	0.000		
Creatinine	1.014 (1.007–1.022)	0.000	1.012 (1.003–1.022)	0.010
FT4	0.926 (0.806–1.064)	0.277		
TSH	0.588 (0.317–1.092)	0.093		
MODS	7.393 (3.395–16.098)	0.000		
ICU admission	12.1 (5.032–27.614)	0.000	4.100 (1.439–11.682)	0.008
Machined ventilation	5.625 (2.435–12.992)	0.000		
Broad-spectrum antibiotic therapy ≥2	10.500 (4.736–23.277)	0.000	5.888 (2.095–16.543)	0.001
**Corticosteroid use**				
Ever	4.020 (1.786–9.048)	0.001	4.631 (1.499–14.312)	0.008
< 1 mg/kg	2.193 (0.877–5.486)	0.093	4.455 (1.207–16.451)	0.025
≥1 mg/kg	11.063 (4.124–29.683)	0.000	4.819 (1.290–18.010)	0.019
≤ 5 d	3.668 (1.515–8.975)	0.004	3.833 (1.134–12.956)	0.031
>5 d	4.556 (1.749–11.869)	0.002	6.470 (1.638–25.556)	0.008

**Figure 1:**
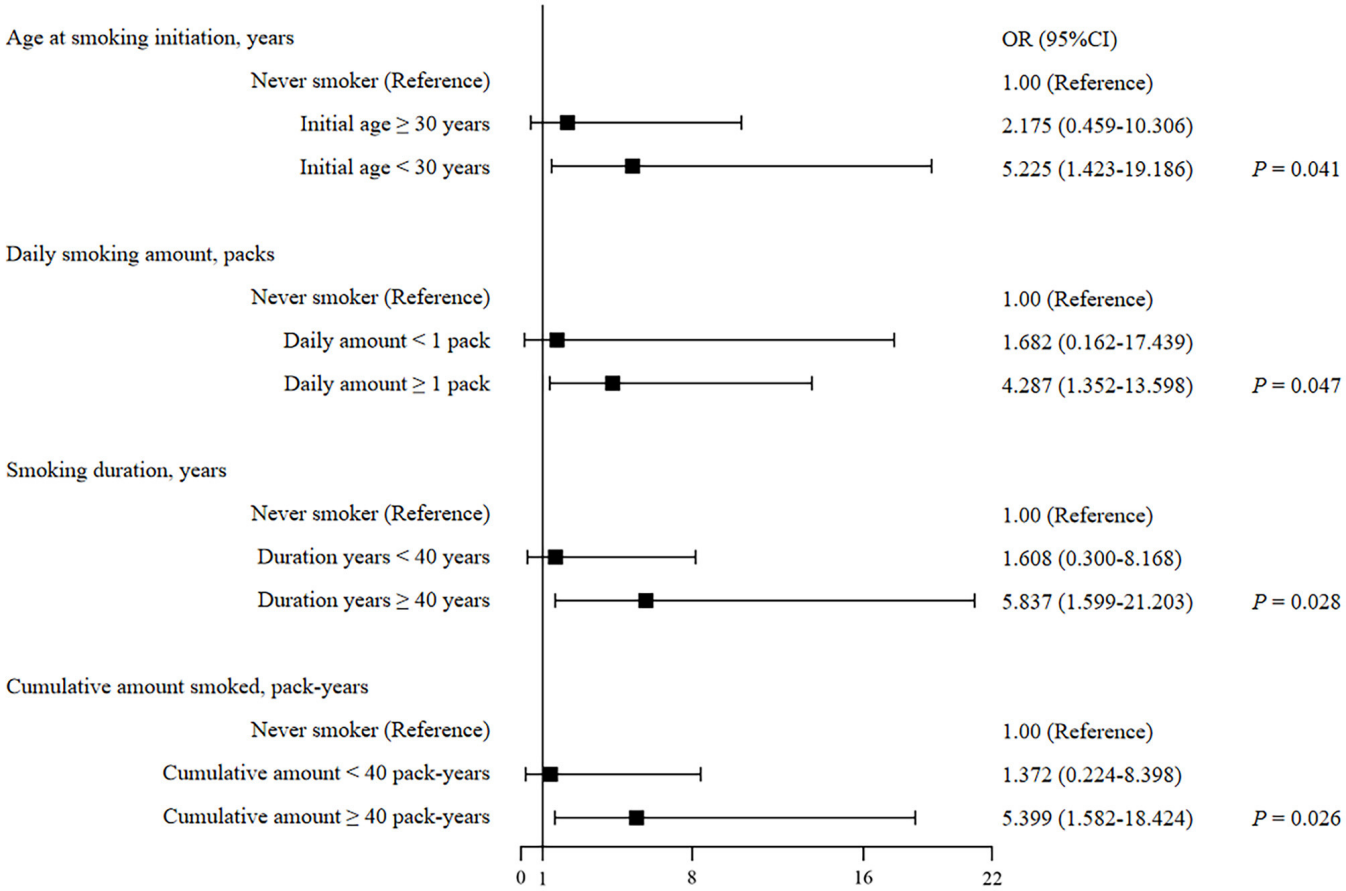
Forest plot of smoking-related factors of predicting IPA development in current smokers with SFTS.

To further assess the relationships among the age of smoking initiation, the daily amount of smoking, smoking duration, pack-years of smoking, and risk of IPA among SFTS patients, a subgroup analysis was performed, which is presented in Table 3 and Figure 1. Of the 39 IPA patients, 20 (51.3%) were ever smokers and 18 (46.2%) were current smokers. Both ever and current smokers had a higher prevalence of IPA when the age for smoking their first cigarette was younger than 30 years (adjusted OR 4.221, 95% CI 1.218–14.631, *P* = 0.023; adjusted OR 5.225, 95% CI 1.423–19.186, *P* = 0.013), while, when the age was at least 30 years, the difference was significant in unadjusted analysis and insignificant in adjusted analysis (adjusted OR 3.275, 95% CI 0.851–12.598, *P* = 0.084; adjusted OR 2.175, 95% CI 0.459–10.306, *P* = 0.328). Our study also illustrated an increase in IPA risk with long smoking duration and high cigarette consumption after adjusting for other major confounding factors. Compared with never smokers, the ever smokers who smoked at least one pack per day had a 3.689-fold risk for the development of IPA, while the current had a 4.287-fold risk (adjusted OR 3.689, 95% CI 1.200–11.338, *P* = 0.023, adjusted OR 4.287, 95% CI 1.352–13.598, *P* = 0.013). The patients who smoked for at least 40 years showed adjusted OR of 5.925 (95% CI 1.629–21.548, *P* = 0.007) for IPA in ever smokers and 5.837 (95% CI 1.599–21.203, *P* = 0.008) in current smokers. The patients with at least 40 pack-years of smoking exposure showed adjusted OR of 5.559 (95% CI 1.696–18.221, *P* = 0.005) in ever smokers and 5.399 (95%CI 1.582–18.424, *P* = 0.007) in current smokers.

**Table 3:** Dose–response between cigarette smoking and the risk of IPA among SFTS patients.

**Variables**	**Ever smoker**					**Current smoker**				
	**IPA/non-IPA**	**Poor OR (95% CI)**	***P-*** **value**	**Adjusted OR (95% CI)**	***P-*** **value**	**IPA/non-IPA**	**Poor OR (95% CI)**	***P-*** **value**	**Adjusted OR (95% CI)**	***P-*** **value**
**Age at smoking initiation**, ***y***										
Never	19/124	1.00 (Reference)		1.00 (Reference)		19/124	1.00 (Reference)		1.00 (Reference)	
≥30	7/12	3.807 (1.332–10.877)	0.013	3.275 (0.851–12.598)	0.084	5/10	3.263 (1.006–10.589)	0.049	2.175 (0.459–10.306)	0.328
< 30	13/14	6.060 (2.473–14.848)	0.000	4.221 (1.218–14.631)	0.023	13/12	7.070 (2.814–17.764)	0.000	5.225 (1.423–19.186)	0.013
*P-*value			0.000		0.040			0.000		0.041
**Daily smoking amount, packs**										
Never	19/124	1.00 (Reference)		1.00 (Reference)		19/124	1.00 (Reference)		1.00 (Reference)	
< 1	4/6	4.351 (1.123–16.853)	0.033	4.018 (0.758–21.306)	0.102	2/4	3.263 (0.559–19.057)	0.189	1.682 (0.162–17.439)	0.663
≥1	16/20	5.221 (2.309–11.803)	0.000	3.689 (1.200–11.338)	0.023	16/18	5.801 (2.533–13.286)	0.000	4.287 (1.352–13.598)	0.013
*P-*value			0.000		0.041			0.000		0.047
**Smoking duration**, ***y***										
Never	19/124	1.00 (Reference)		1.00 (Reference)		19/124	1.00 (Reference)		1.00 (Reference)	
< 40	6/16	2.447 (0.852–7.030)	0.316	2.266 (0.582–8.827)	0.238	4/12	2.175 (0.636–7.445)	0.216	1.608 (0.300–8.168)	0.579
≥40	14/10	9.137 (3.554–23.491)	0.000	5.925 (1.629–21.548)	0.007	14/10	9.137 (3.554–23.491)	0.000	5.837 (1.599–21.203)	0.008
*P-*value			0.000		0.023			0.000		0.028
**Cumulative amount smoked, pack-years**										
Never	19/124	1.00 (Reference)		1.00 (Reference)		19/124	1.00 (Reference)		1.00 (Reference)	
< 40	5/12	2.719 (0.861–8.585)	0.088	1.698 (0.351–8.205)	0.510	4/9	2.901 (0.812–10.359)	0.101	1.372 (0.224–8.398)	0.732
≥40	15/14	6.992 (2.918–16.755)	0.000	5.559 (1.696–18.221)	0.005	14/13	7.028 (2.869–17.220)	0.000	5.399 (1.582–18.424)	0.007
*P-*value			0.000		0.018			0.000		0.026

### Predictive values of the risk factors for IPA

**Figure 2:**
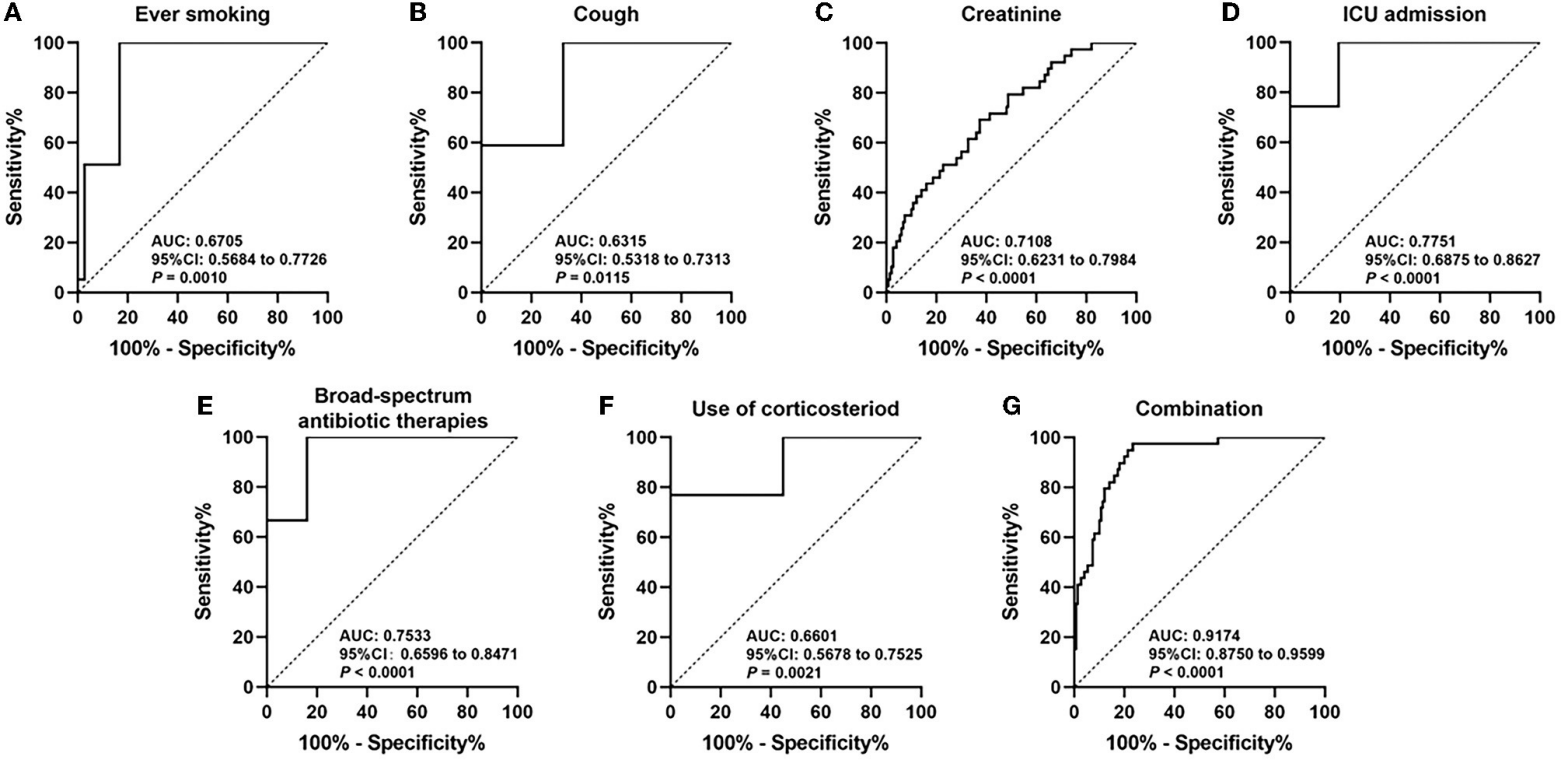
Receiver operating curve (ROC) analyses of ever smoking **(A)**, cough **(B)**, creatinine **(C)**, ICU admission **(D)**, broad-spectrum antibiotic therapies **(E)**, use of corticosteroid **(F)**, and combination of all risk factors **(G)** in estimating the risk of IPA development in SFTS patients.

Figure 2 shows the ROC curves of each independent risk factor, and the combination of all risk factors based on the multivariate analysis model is shown in Table 2. The AUCs of ever smoking, cough, creatinine, ICU admission, the combination of several broad-spectrum antibiotic therapies, and use of corticosteroid to predict the development of IPA in SFTS patients were 0.6705 (95% CI 0.5684–0.7726, *P* = 0.0010), 0.6315 (95% CI 0.5318–0.7313, *P* = 0.0115), 0.7108 (95% CI 0.6231–0.7984, *P* < 0.0001), 0.7751 (95% CI 0.6875–0.8627, *P* < 0.0001), 0.7533 (95% CI 0.6596–0.8471, *P* < 0.0001), and 0.6601 (95% CI 0.5678–0.7525, *P* = 0.0021). Furthermore, the AUC–ROC increased to 0.9174 (95% CI 0.8750–0.9599, *P* < 0.0001) when they were combined to predict the IPA in SFTS patients.

### Survival time of the IPA group and the non-IPA group

**Figure 3:**
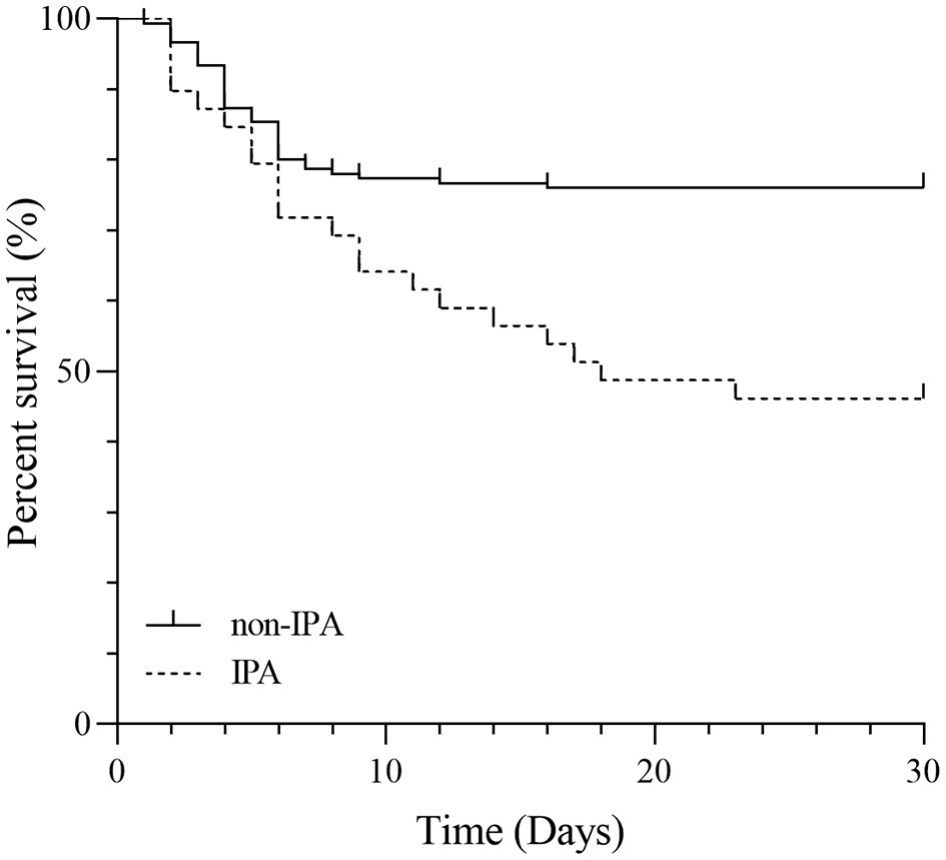
Kaplan–Meier curves for SFTS patients in the IPA and non-IPA groups.

For these SFTS patients, the mortality rates were 53.8% for the IPA group and 24.0% for the non-IPA group with a significant difference (*P* < 0.05). Figure 3 shows the comparison of survival curves between the two groups. The non-IPA group always had a lower mortality probability than the IPA group during the 1-month follow-up period. The difference in mortality rate increased sharply after the first week.

## Discussion

Given the high popularity and mortality of IPA development in SFTS patients, we performed a study with the highest number of cases to date to evaluate the risk factors (Seong et al., 2022). Meanwhile, this is the first study to reveal strong evidence of the correlations between the status of tobacco smoking exposure and IPA development in SFTS cases. Our survey showed that cough, high level of creatinine on admission, ICU admission, treatment with a combination of several antibiotic therapies, or treatment with corticosteroids were significant risk factors for IPA development. Our results also demonstrated that smoking was an important risk factor for IPA development, with dose-dependent effects on several indicators of smoking exposure.

Smoking has been identified as a modifiable lifestyle habit that can affect humoral and cellular immune responses. It can mainly raise a disruption to the respiratory system by inducing inflammation and damage to the pulmonary epithelia and inhibiting mucociliary clearance. In addition to containing a complex mixture of various chemicals that has a regulating role for the immune system, cigarettes were found to contain multiple types of fungal spores, of which *Aspergillus fumigatus* accounted for the largest proportion (Chien et al., 2019; Chattopadhyay et al., 2021; Oosterwijk et al., 2023). Therefore, spores of Aspergillus could make it all the way to the lungs. All of these factors combine to provide the maximum probability that smoking has a strong association with IPA. Various studies have proved that patients with avian influenza A (H7N9), influenza, or COVID-19 who had a history of smoking had increased risks of IPA vs. non-smokers (Zou et al., 2020; Calderón-Parra et al., 2022; Shi et al., 2022). Contrary to these findings, a meta-analysis published in 2021 has highlighted the role of smoking in increasing the risk of invasive fungal disease development; however, the relationship between IPA and smoking was found to be non-significant (Pourbaix et al., 2020). In our multivariable model, current smokers had a 3.764-fold increased risk of developing IPA. In addition, previous studies presented that smoking resulted in damage to the kidney, and our findings found that renal damage might further increase the risk of IPA (Oosterwijk et al., 2023). We also demonstrated that there was a dose-response relationship between exposure to cigarette smoking and the occurrence of IPA. In a subgroup analysis, no matter in ever smokers or current smokers who had long smoking duration, high cigarette consumption, and great cumulative exposure increased the risk of IPA development by ~5-fold. Taken together, SFTS patients with a smoking history tend to have an increased risk of IPA development, especially for those with high-dose exposure to smoking.

From the perspective of laboratory indicators, high viral load has been revealed as one of the indicators of poor prognostic for SFTS in several studies, but studies performed to explore the association between viral RNA loads of DBV and the occurrence of IPA are currently lacking (Wei et al., 2022). Bae et al. (2020) reported that the viral load level was significantly higher in the IPA group, which corresponded with our result but did not go into more detail via regression analysis. For the present study, high viral load showed a significant relationship with IPA in univariate analysis and lost relevance in multivariate analysis, pointing out the index's limitation on risk assessment. In addition, similar to previous studies which confirmed that the risk of IPA increased significantly in SFTS patients with nephritic damage, we further demonstrated it by showing the strong association between a high level of creatinine and the risk of IPA in SFTS cases (Hu et al., 2021).

Inflammatory cytokine storm is an excessive immune response, which is characterized by elevated levels of proinflammatory cytokines and chemokines and has recently been considered to correlate with the worse outcome in SFTS patients (Yoo et al., 2021). In our study, we also found that compared with non-IPA patients, IPA patients had higher levels of several cytokines, including IL-6, IL-8, IL-10, IL-17, IFN-α, and IFN-γ (data not shown), emphasizing the exaggerated systemic inflammation in IPA patients. Only a few SFTS patients were tested with these indicators, which makes us unable to further explore the correlations between these cytokines and the development of IPA. However, we reported other inflammatory and immunological markers of SFTS patients, such as serum ferritin, LDH, ADA, IL-6, lymphocyte counts, and subsets. Collectively, we noticed that ferritin, LDH, and IL-6 were higher in IPA patients, indicating a sign of severe inflammation and immune injury in these patients. Of note, Hu et al. demonstrated that counts of CD4^+^ T-cells < 68 cells/mm^3^ combined with CD8^+^ T-cells < 111 cells/mm^3^ were independent risk factors for the development of IPA in SFTS patients, and Song et al. demonstrated that CD4^+^ T-cells < 300 cells/μL and CD8^+^ T-cells < 400 cells/μL were also independent risk factors for IPA. However, there was no significant difference in lymphocyte subsets between the two groups in our study, which might be due to the small sample size and individual differences (Hu et al., 2021; Song et al., 2023).

Our study showed the frequencies of certain therapy-related factors and determined their role in developing IPA. After adjusting potential confounding variables, admission to ICU, combination of several broad-spectrum antibiotic therapies, and use of corticosteroids resulted in higher chances for IPA development. Corticosteroid usage can not only alleviate leukocyte activities and phagocytosis function but also diminish the inhibition effects of pulmonary macrophages on the growth of Aspergillus (Jung et al., 2021; Seong et al., 2021; White et al., 2021). This study established that SFTS patients who were treated with large doses or long-term use of corticosteroids were more likely to develop IPA. Moreover, a case–control study in Japan also reported that all SFTS cases with IPA were in the corticosteroid-treated group (Kawaguchi et al., 2021). Meanwhile, more than or equal to two antibacterial drugs used together can lead to growing drug resistance, raising the reproduction speed of Aspergillus. Various invasive operations such as mechanical ventilation are effective treatments for patients with breathlessness, which may cause injury to the epithelial cells and increase the risk of IPA development while no relevance between this operation and IPA was found in the multi-factor model of our study. Therefore, we should treat patients with antibiotics and corticosteroid therapies with caution and take into consideration the possibility of IPA when they present with suspicious symptoms.

Several mechanisms have been suggested to explain the susceptibility of IPA in SFTS patients. For one thing, reduced cellular and humoral immune function might contribute to the increased secondary infections of SFTS patients (Kim et al., 2020a,b; He et al., 2021). Furthermore, research has speculated that tracheal intubation, an important treatment to maintain the patient's airway in severe SFTS cases, can diminish respiratory epithelial cells' capacity for adherence and germination, which could facilitate fungi invasion into the immune-compromised patients (Chen et al., 2018). Furthermore, thrombocytopenia is a typical symptom of SFTS, which may lead to a decrease in the ability of platelets to block the hyphal elongation of *Aspergillus fumigatus* (Speth et al., 2013). In our study, 20.6% of the SFTS patients were diagnosed with IPA, among whom 53.8% were dead with a significantly shorter survival time. Consistent with our results, two retrospective studies showed high CFRs of 53.3 and 44.4% in the IPA group, which indicated the urgency of earlier detection, diagnosis, and intervention. Theoretically, antifungal prophylaxis can reduce the risk of IPA development in SFTS by blocking the colonization of Aspergillus, which remains to be explored due to the limited data.

Our study had several limitations. First, the status of smoking originated from self-reports of the patients possibly contributing to an inherent bias. Second, smoking includes exposure to active, passive, and third-hand smoking; our study focused on active smoking, but the effect of the two remaining types could not be evaluated. Third, as a retrospective study, our study was unable to have a real-time observation of the patients.

In conclusion, our study provided evidence of the high incidence and mortality of IPA in SFTS patients. For this emerging infectious disease without effective treatments or vaccines, identifying risk factors associated with this lethal complication is critical. Attention should be paid to SFTS patients with independent risk factors, including smoking history, cough, high level of creatinine, admission to ICU, and treatment with a combination of several antibiotic therapies or corticosteroids. With regard to smoking, our study suggests it to be an important, dose-dependent risk factor for IPA development. Our findings also provide the insight that further research is needed to determine if antifungal prophylaxis can prevent IPA development or improve the prognosis in SFTS patients.

## Data availability statement

The original contributions presented in the study are included in the article/supplementary material, further inquiries can be directed to the corresponding authors.

## Author contributions

YD and QP: study conception and design and language editing. YD, QP, NH, YH, and PS: primary data collection and data analysis. JZ: review of the initial manuscript. JL and KJ: funding acquisition and supervision. All authors revised the manuscript for the main content and approved the final manuscript for publication.
